# Treatment of growth hormone attenuates hepatic steatosis in hyperlipidemic mice via downregulation of hepatic CD36 expression

**DOI:** 10.1080/19768354.2020.1778080

**Published:** 2020-06-12

**Authors:** Hyung Seok Jang, Kyeongdae Kim, Mi-Ran Lee, Shin-Hye Kim, Jae-Hoon Choi, Mi Jung Park

**Affiliations:** a Department of Life Science, College of Natural Sciences and Research Institute for Natural Sciences, Hanyang University, Seoul, Republic of Korea; b Department of Biomedical Laboratory Science, Jungwon University, Goesan, Republic of Korea; c Department of Pediatrics, Inje University Sanggye Paik Hospital, Seoul, Republic of Korea

**Keywords:** Growth hormone, hyperlipidemia, hepatic steatosis, atherosclerosis, CD36

## Abstract

The recombinant human growth hormone (GH) has been used for the treatment of growth hormone deficiency (GHD) and diverse short stature state, and its physiological and therapeutic effects are well documented. However, since the effect of GH treatment on metabolic disorders has not been well characterized, we injected GH to Western diet-fed low-density lipoprotein receptor-deficient (*Ldlr*^−/−^) mice to understand the exact effect of GH on metabolic diseases including atherosclerosis, hepatic steatosis, and obesity. Exogenous GH treatment increased plasma IGF-1 concentration and decreased body weight without affecting serum lipid profiles. GH treatment changed neither atherosclerotic lesion size nor collagen and smooth muscle cells accumulation in the lesion. GH treatment reduced macrophage accumulation in adipose tissue. Importantly, GH treatment attenuated hepatic steatosis and inflammation. The hepatic expression IL-1β mRNA were decreased by GH treatment. The mRNA and protein levels of CD36 were markedly decreased in GH treated mice without significant changes in other molecules related to lipid metabolism. Therefore, the treatment of GH treatment could attenuate hepatic steatosis and inflammation with downregulation of CD36 expression in hyperlipidemic condition.

## Introduction

Growth hormone, a 22 kDa polypeptide produced in the anterior pituitary, is a potent metabolic modulator. It plays an important role in the immune system, in addition to its established roles in tissue growth and metabolism (Kelley et al. 2007). GH promotes growth in childhood and adolescence by stimulating various types of cells and tissues, such as the bone, skin, muscle, immune cells, and cartilage (Caicedo et al. 2018). The GH and insulin-like growth factor-1 (IGF-1) axis play a central role in the growth and development of organ systems. GH does not act alone to stimulate mitosis and differentiation in all tissues. The liver expresses a GH-specific receptor, and hepatocytes produce IGF-1 after activation of the GH receptor. GH and IGF-1 promote growth and function in many tissues (Kargi and Merriam 2013).

While GH induces lipolysis, the lipogenesis in adipose tissue is inhibited by GH, leading to significant fat loss and increased muscle mass (Fisker et al. 1998; Rasmussen 2010). It has been reported that the IGF-1 level was lower in obese individuals compared to normal counterparts, suggesting an inverse relationship between GH/IGF-I axis and fat mass distribution (Rasmussen et al. 1994; Juul 2003).

Nonalcoholic fatty liver disease (NAFLD) is recognized as the most common cause of chronic liver disease worldwide. NAFLD is classified into the non-alcoholic fatty liver (NAFL) and non-alcoholic steatohepatitis (NASH), and its symptoms may vary from simple steatosis to liver cirrhosis (Angulo 2002; Hashimoto and Tokushige 2011). Previously, it has been reported that NAFLD is more common in hypopituitary patients having impaired secretory function of GH (Adams et al. 2004; Hong et al. 2011). Thus, GH deficiency and the development of NAFLD/NASH may be closely related. In addition, low IGF-1 levels in patients with chronic NAFLD patients indicate that GH resistance may be associated with the severity of the disease (Chishima et al. 2017). Importantly, it has been reported that GH replacement therapy for adult GH deficiencies (GHD) significantly reversed NASH, while reducing the markers of colorectal cancer and oxidative stress (Takahashi et al. 2007).

Previously, it has been reported that adult GHD patients have increased cardiovascular morbidity and mortality (Di Somma et al. 2017). GHD is associated with an increased risk of atherosclerosis (Binay et al. 2015). In this context, endothelial dysfunction is reversed by recombinant human GH therapy in patients with GHD, which may reduce the risk of developing atherogenesis (Graham et al. 2009). In addition, cardiovascular risk in GHD adults, such as changes in body composition, abnormal lipid profile and glucose metabolism, and insulin resistance, can be partially reduced by GH replacement therapy (Di Somma et al. 2017). Therefore, GH treatment may be a good therapeutic option for treating atherosclerosis.

Collectively, it appears that GH may play pivotal roles in inflammatory metabolic disorders, including obesity, NAFLD, and atherosclerosis. These metabolic diseases share many risk factors and can affect their progression each other. Thus it is important to analyze the exact effects of GH treatment on the progression of these metabolic diseases in an animal model having these metabolic diseases. In this study, we investigated the effects of GH on the pathological changes of aorta, liver, and fat tissue in Western diet-fed Ldlr-/- mice susceptible to atherosclerosis, NAFLD, and obesity.

## Materials and methods

### Mice

*Ldlr^-/-^* mice were obtained from Jackson Laboratory (Bar Harbor, ME, USA). The mice were bred in a specific pathogen-free facility under 12 h light and 12 h dark cycle, with free access to food and water. Fifteen male *Ldlr^-/-^* mice (10 week old) were fed a western diet (WD) from Test Diet (AIN-76A; St. Louis, MO, USA) for 10 weeks. All animal experiments were performed with the approval of the Institutional Animal Care and Use Committee of Hanyang University, Seoul, Korea.

### Recombinant human growth hormone injection

GH (Eutropin®) was obtained from LG Chem. Ltd. (Seoul, Korea) and diluted with phosphate-buffered saline (PBS). GH (0.33 g/kg per body weight) or PBS (200 µl) were injected subcutaneously to *Ldlr^-/-^* mice (vehicle group, n=7; GH treated group, n=8) every day at 10 AM for 10 weeks.

### Assessment of atherosclerosis lesion

For the aortic sinus section, hearts were perfused with cold PBS and fixed with 4% paraformaldehyde. Four-micrometer-thick cryosections were prepared and hydrated with PBS, and stained with Oil red O, hematoxylin or picrosirius red. The specimens were observed under an Olympus BX54 and photographed with DP74. The lesions were analyzed using the Olympus CellSens Software.

### Assessment of blood lipid profile and IGF-1 level

Plasma lipid levels were analyzed using an automated blood chemical analyzer (Hitachi, Tokyo, Japan). Plasma IGF-1 levels were analyzed using the mouse IGF-1 enzyme-linked immunosorbent assay (ELISA) kit (cat. ab100695, Abcam, Boston, MA, USA) according to the manufacturer’s protocol.

### Immunohistochemistry (IHC)

The frozen sections were immunostained with anti-macrophage/monocyte antibody (MOMA-2) After washing with PBS, the sections were reacted with Alexa Fluor 488 (goat anti-mouse Ig) and washed. The tissues were reacted with Cy3-conjugated anti-α-smooth muscle antibody (SMA) for 1h at RT, washed thrice with PBS, and then mounted with 4′,6-diamidino-2-phenylindole (DAPI) mounting solution (Vector Labs, Burlingame, CA, USA).

### Flow cytometry

For single-cell isolation, the adipose tissues were minced to 2 − 4-mm pieces and incubated with collagenase II solution (400 U/ml, cat. C6885; Sigma-Aldrich) for 8 min. To eliminate dead cells, the cells were pre-stained with live dead staining antibody (Zombie Aqua; cat.423101; BioLegend). The Fc receptors were blocked to reduce non-specific binding to the receptors, and the cells were stained with flow cytometry antibodies at 4°C for 30 min. The cells were gently washed with 2% fetal bovine serum (FBS) in PBS and read using a fluorescence-associated cell sorter (FACS) LSR II or Canto II instrument (BD Biosciences, Franklin Lakes, NJ, USA). The data were analyzed using FlowJo software (Tree Star Inc, Ashland, OR, USA).

### Measurement of hepatocellular steatosis and inflammation

The percentages (%) of macrovesicular and microvesicular steatosis were scored based on the total area. The criteria for the identification of macrovesicular steatosis and microvesicular steatosis was based on whether the nuclei were displaced peripherally by lipid droplets (macrovesicular steatosis) or not (microvesicular steatosis), as well as by whether the lipid droplet existed as one large vacuole (macrovesicular steatosis) or several small vacuoles (microvesicular steatosis). All steatosis was evaluated at 40∼100× magnification, and only the hepatocytes were measured. At 100× magnification, the inflammatory cells were counted at 10 different fields, and the average number of the cells was calculated. The lesions were analyzed under an Olympus BX54 microscope and photographed using DP74.

### Western blot analysis

The separated proteins in a 10% SDS-polyacrylamide gel were blotted on a polyvinylidene fluoride (PVDF) membrane. The primary and secondary antibodies were monoclonal anti-CD36 antibody (Santa Cruz, cat. sc-7309) and 1:10,000 diluted horseradish peroxidase conjugated anti-mouse antibody (Thermo Scientific, cat. 31430), respectively.

### Quantitative reverse transcription polymerase chain reaction (qRT-PCR)

qRT-PCR was performed using the Rever TraACE® qPCR RT master mix (Toyobo Co., Osaka, Japan) according to the manufacturer's instructions. qRT–PCR was performed in three-steps using Rotor-gene Q (Qiagen, Hilden, Germany) and SYBR Green PCR master mix (KAPA Biosystems, Wilmington, MA, USA). The specific primer pairs used are listed in Table 1.

**Table 1.** Primer sequences.

*Actb1* (β-actin)	Forward	5′-ACGGCCAGGTCATCACTATTG-3′
	Reverse	5′-CACAGGATTCCATACCCAAGAAG-3′
*Il1b*	Forward	5′-GGAGAACCAAGCAACGACAAAATA-3′
	Reverse	5′-TGGGGAACTCTGCAGACTCAAAC-3′
*Il6*	Forward	5′-CCAGAGATACAAAGAAATGATGG-3′
	Reverse	5′-ACTCCAGAAGACCAGAGGAAAT-3′
*Tnfa*	Forward	5′-TGGCCCAGACCCTCACACTCAG-3′
	Reverse	5′-ACCCATCGGCTGGCACCACT-3′
*Nos2*	Forward	5′-CCCTTCAATGGTTGGTACATGG-3′
	Reverse	5′-ACATTGATCTCCGTGACAGCC-3′
*Cd206*	Forward	5′-TCTTTG CCTTTCCCAGTCTCC-3′
	Reverse	5′-TGACACCCAGCGGAATTTC-3′
*Il10*	Forward	5′-GCTCCAAGACCAAGGTGTCT-3′
	Reverse	5′-CTAGGTCCTGGAG TCCAGCA-3′
*Chil3*	Forward	5′-CATGATCCTAAGGATGGCTAC-3′
	Reverse	5′-CAATGAGCTTCTCAGAAGCTG-3′
*Arg1*	Forward	5′-GAACACGGCAGTGGCTTTAAC-3′
	Reverse	5′-TGCTTAGTTCTGTCTGCTTTGC-3′
*Cd36*	Forward	5′-GTTCTTCCAGCCAATGCCTTT-3′
	Reverse	5′-GTTCTTCCAGCCAATGCCTTT-3′
*Srebp1c*	Forward	5′-GCTGTTGGCATCCTGCTATC-3′
	Reverse	5′-TAGCTGGAAGTGACGGTGGT-3′
*Fabp1*	Forward	5′-TATGGACCCAAAGTGGTCCG-3′
	Reverse	5′-AGCTTGACGACTGCCTTGAC-3′
*Mttp*	Forward	5′-GTGCCATGCAAAATAGCGGT-3′
	Reverse	5′-TCTTGCGGTTTTCCTTTGCC-3′
*Apob100*	Forward	5′-AGCACCTCCGAAAGTACGTG-3′
	Reverse	5′-TCTTGCGGTTTTCCTTTGCC-3′
*Ppara*	Forward	5′-GAAAGACCAGCAACAACCCG-3′
	Reverse	5′-TCTTTGTCTTCGACGCCGTT-3′
*Cyp7a1*	Forward	5′-GCTGAGAGCTTGAAGCACAAGA-3′
	Reverse	5′-TTGAGATGCCCAGAGGATCAC-3′
*Cyp8b1*	Forward	5′-GCCCACAGCCTTCAAGTATG-3′
	Reverse	5′-CGACCAGCTTGAAGTCGAAG-3′
*Srebp2*	Forward	5′-GCGCCAGGAGAACATGGT-3′
	Reverse	5′-CGATGCCCTTCAGGAGCTT-3′
*Sra1*	Forward	5′-TTTGGAACAGGCATTGGAAG-3′
	Reverse	5′-GCGGTGGATGTCATCTGCT-3′
*Abca1*	Forward	5′-TGAAGCCTGTCCAGGAGTTC-3′
	Reverse	5′-ATGACAAGGAGGATGGAAGC-3′
*Abcg1*	Forward	5′-CAAGACCCTTTTTGAAAGGGATCTC-3′
	Reverse	5′- GCCAGAATATTCATGAGTGTGGAC -3′

### Statistical analysis

Most of data were shown as mean ± standard deviation; qRT-PCR data were shown as mean ± standard error of the mean. We conducted a normality test (Shapiro-Wilk) to determine whether the values satisfied the normality assumption. Two-group independent t-test was used for parametric analysis; otherwise Mann-Whitney test was used for nonparametric analysis. Statistical analyses were performed using Prism 6 (GraphPad software, La Jolla, CA, USA) or Instat software (GraphPad software).

## Results

### GH treatment increased serum IGF-1 level

**Figure 1.**
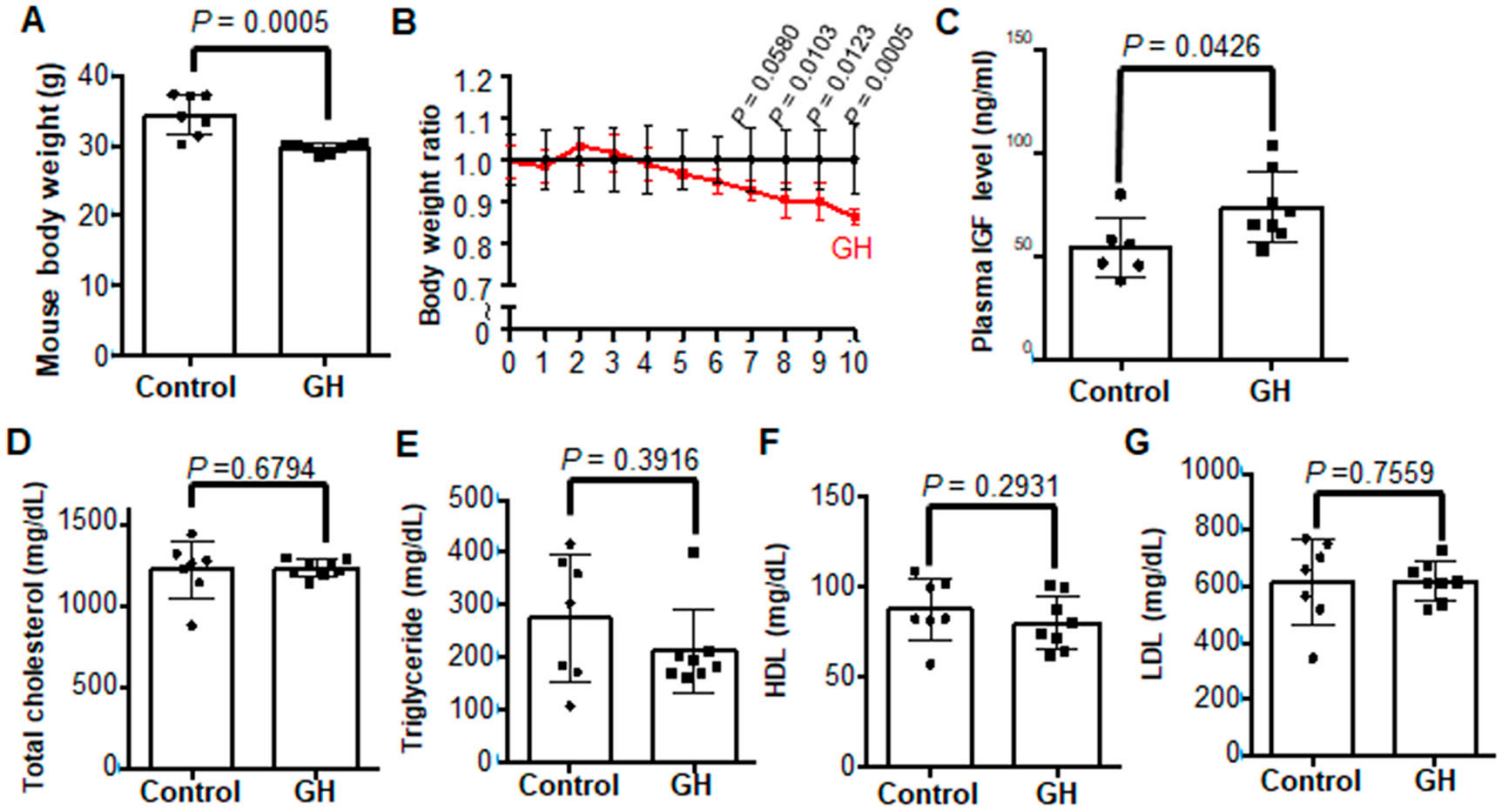
Effect of GH treatment on body weights, plasma IGF concentrations and lipid profiles. (A, B) The body weights were significantly reduced in GH treated *Ldlr^-/-^* mice compared to control mice. (C) Plasma IGF concentration was significantly increased in the GH-treated group. (D-G) Total plasma cholesterol, triglycerides, HDL, and LDL were not changed by GH treatment for 10 weeks.

The body weight decreased significantly in the GH-treated *Ldlr^-/-^* mice compared to the vehicle-treated *Ldlr^-/-^* mice after 10 weeks (Figure 1(A and B)). Plasma IGF concentration was significantly increased by GH treatment (Figure 1(C)). But the lipid concentrations were not changed by GH treatment. The concentrations of total cholesterol, triglycerides, HDL, and LDL in GH treated mice were comparable to those of control mice (Figure 1(D–G)).

### GH treatment did not affect atherosclerotic lesion formation

**Figure 2.**
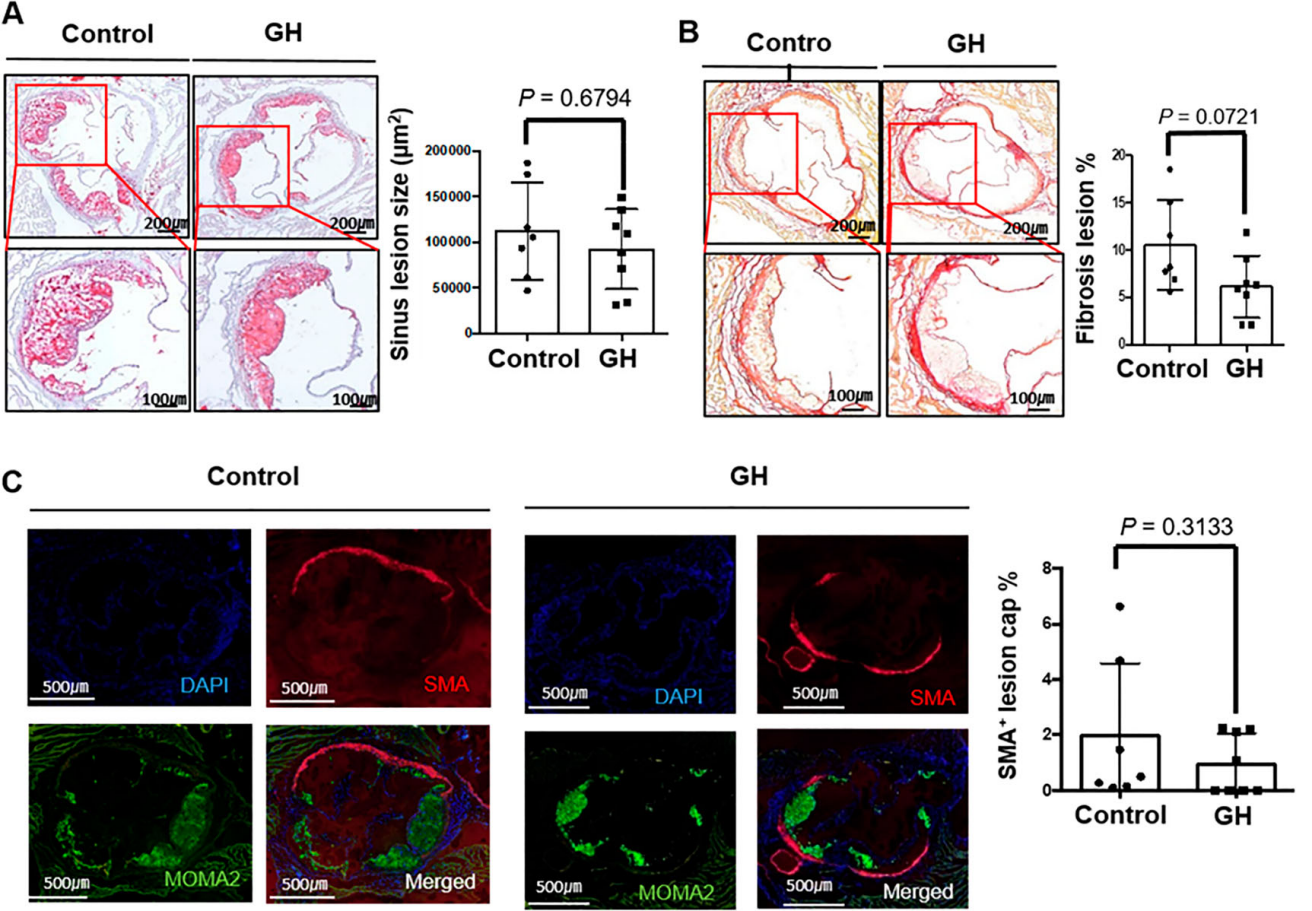
Effect of GH treatment on atherosclerotic lesion formation. (A) The representative figures of Oil red O-positive atherosclerotic lesions and lesion size in each group. (B) The degree of collagen accumulation in the lesion was evaluated using Picro-sirius Red staining. (C) Immunofluorescence staining for smooth muscle cells (SMA) and macrophages (MOMA-2). The content of smooth muscle cells in atherosclerotic lesion was not changed by GH treatment.

Although the plasma lipid levels were not significantly different between the GH-treated and control groups, the severity of atherosclerosis was assessed by the measurement of Oil Red O stained lesion size. And the lesion size was not changed by GH treatment (Figure 2(A)). To further characterize the plaque phenotype, Picro-sirius red staining was performed to analyze the collagen accumulation in the lesions. The lesional collagen accumulation was not affected by GH treatment (Figure 2(B)). Lesional **s**mooth muscle cells and macrophages were immunostained using anti-smooth muscle actin (SMA) and MOMA2 antibodies. The SMA positive area was not significantly changed by GH treatment (Figure 2(C)).

### GH treatment decreased the number of macrophages in adipose tissue

**Figure 3.**
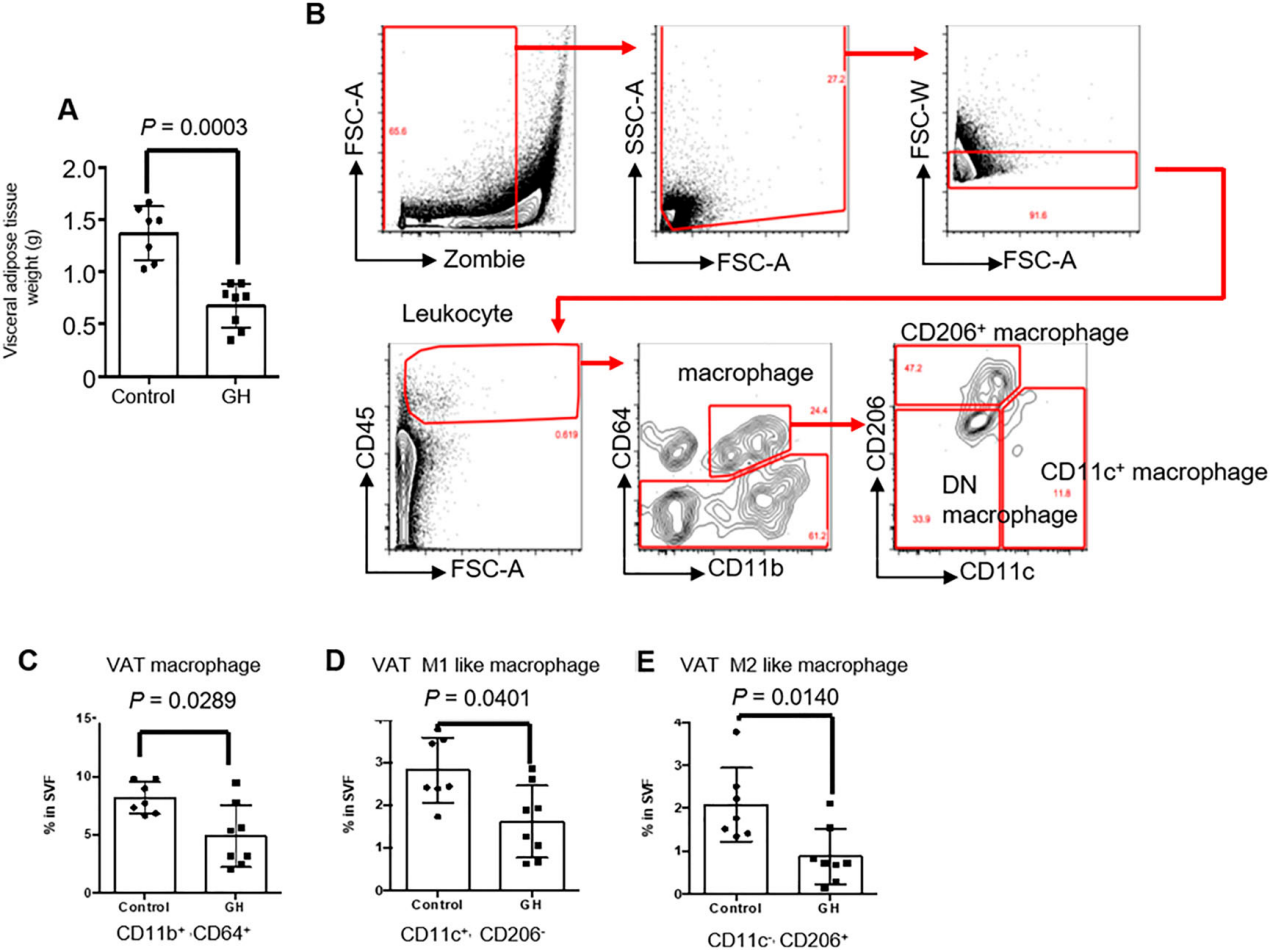
Effect of GH treatment on adipose tissue macrophages. (A) Weight of visceral adipose tissue. (B) Gating strategy for flow cytometric analysis of adipose tissue macrophages. (C-E) The percentages of total macrophages (CD11b^+^ CD64^+^), M1-like macrophages (CD11c^+^ CD206^−^), and M2-like macrophages (CD11c^−^ CD206^+^) in visceral adipose tissue.

GH treatment markedly decreased the weight of visceral adipose tissue (Figure 3(A)). Thus, we analyzed adipose tissue macrophages that have a pivotal role in fat inflammation leading to metabolic diseases. First, the macrophages (CD11b^+^CD64^+^) were divided into three populations including CD206^+^ M2-like macrophage, CD11c^+^ M1-like macrophage and CD11c^-^CD206^-^ double negative (DN) macrophage (Figure 3(B)). The number of total VAT macrophages (CD11b^+^CD64^+^), VAT M1-like macrophages (CD11c^+^CD206^−^), and VAT M2-like macrophages (CD11c^−^CD206^+^) (Figure 3(C–E)) were significantly decreased in the GH-treated group.

### GH treatment attenuates hepatic steatosis and inflammation

**Figure 4.**
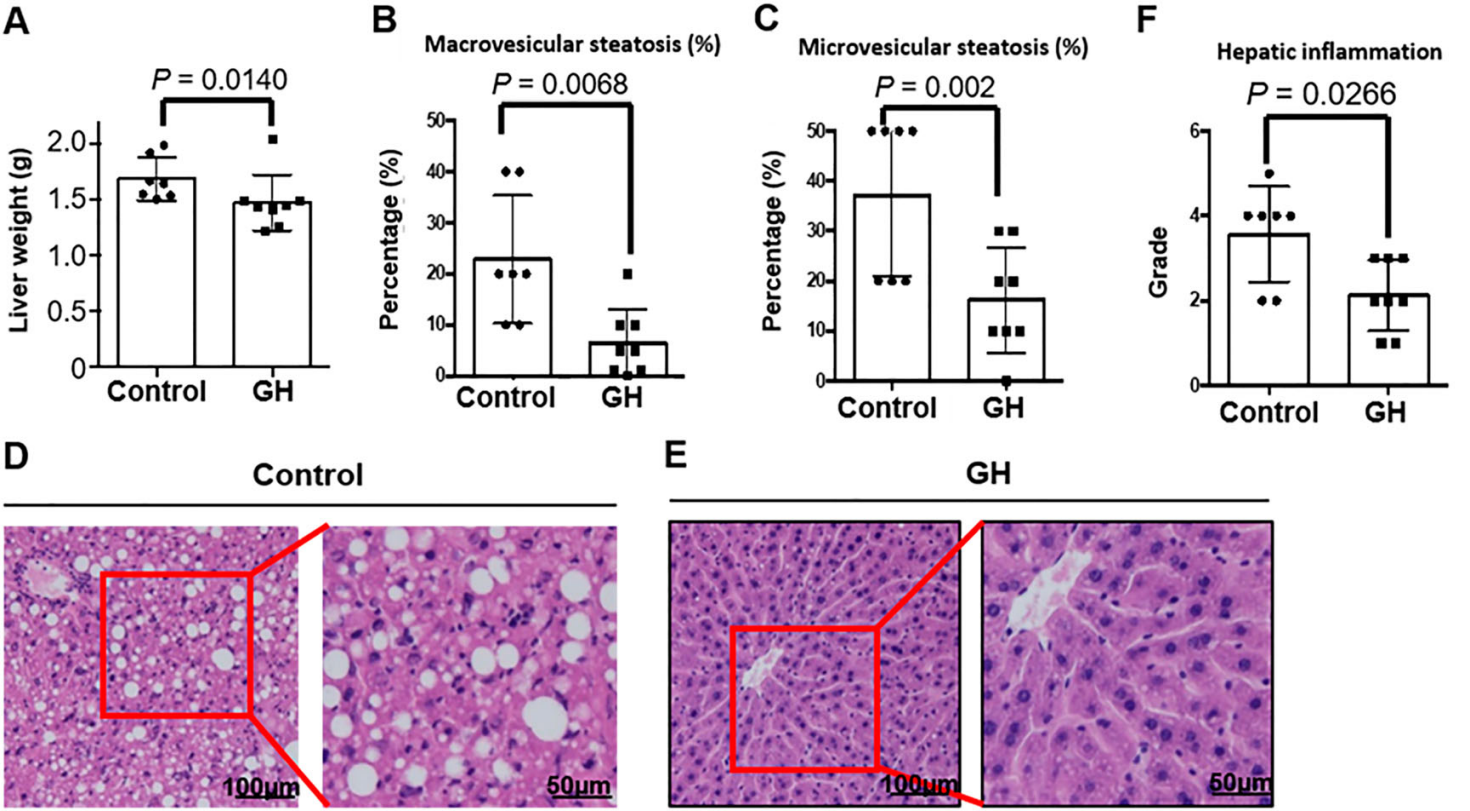
Effects of GH treatment on hepatic steatosis and inflammation. (A) Weights of liver. (B, C) The percentages of macrovesicular steatosis and microvesicular steatosis in hepatic tissue. (D, E) Representative histological figures from control and GH treated groups. (F) Hepatic inflammation score was significantly reduced in the GH-treated group compared to control.

**Figure 5.**
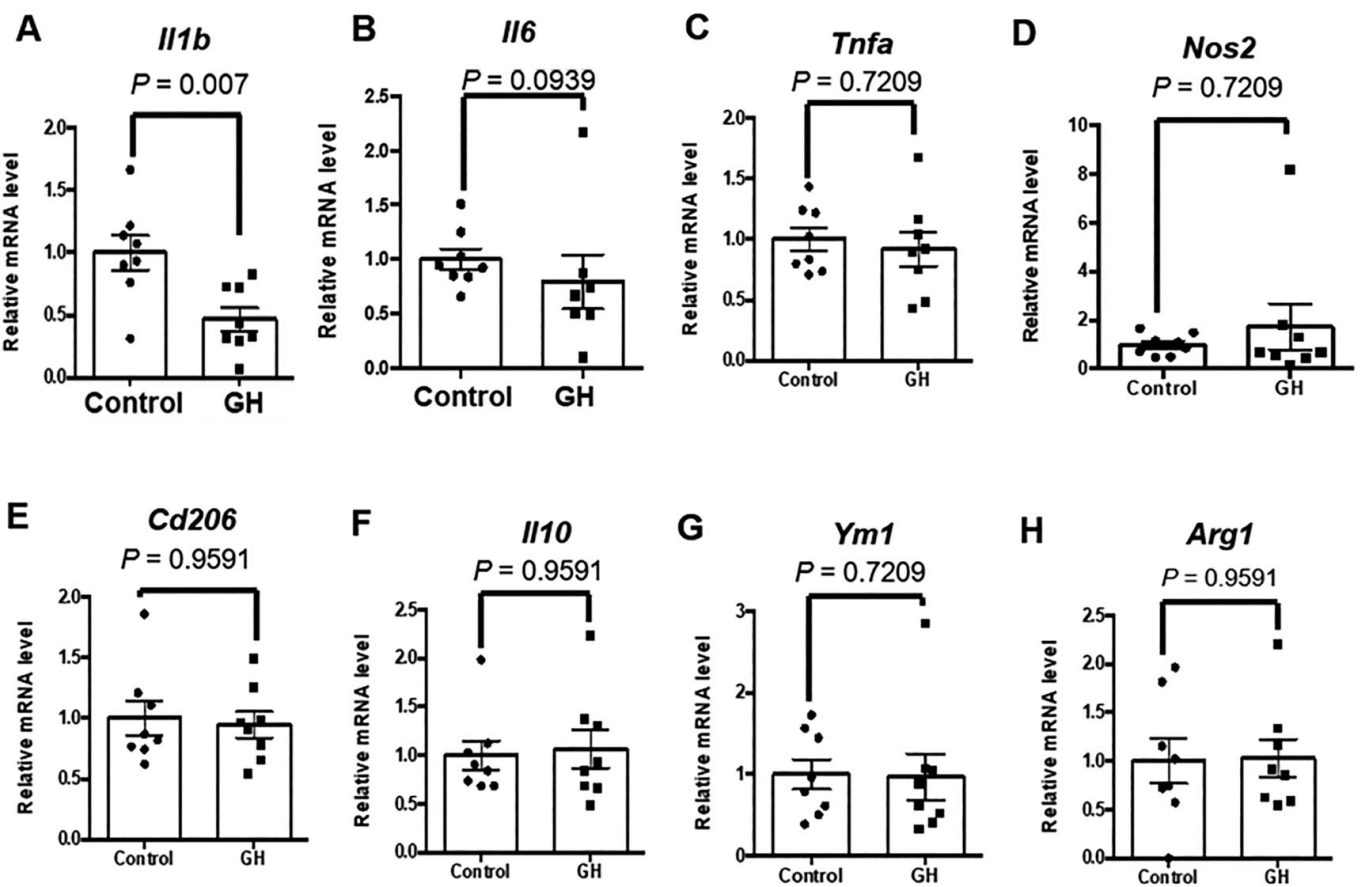
Effects of GH treatment on hepatic gene expressions related to inflammation. Hepatic gene expressions related to inflammation were analyzed by quantitative RT-PCR. (A) IL-1β, (B) IL-6, (C) TNF-α, (D) Nos2, (E) CD206, (F) IL-10, (G), YM1, and (H) Arginase-1 mRNA expressions. Hepatic *Il1β* expression was markedly decreased by GH treatment.

Liver weights were significantly reduced in GH-treated mice (Figure 4(A)). Hence, we investigated whether GH treatment affected hepatic steatosis and inflammation. In the control group, large amounts of fat accumulated in hepatocytes as a form of lipid vacuoles. Hepatic steatosis and steatohepatitis were also observed. There were macrovesicular foci and inflammatory cells, including lymphocytes and macrophages in the hepatic lobules. However, the number of lipid droplets in the GH-treated group was markedly decreased, and the degree of macrovesicular and microvesicular steatosis was significantly decreased by GH treatment (Figure 4(B–E)). Hepatic inflammatory changes including micro-foci of lymphocytes were significantly attenuated in the GH-treated group (Figure 4(F)). These results prompted us to investigate the changes in the mRNA levels of inflammatory and anti-inflammatory factors. The IL-1β level was lower in the GH-treated group than in control (Figure 5(A)). However, there were no significant differences between the two groups in terms of IL-6, TNF-α, iNOS, CD206, IL-10, YM1, and Arginase-1 mRNA levels (Figure 5(B–H)).

### GH treatment markedly decreased hepatic CD36 expression

**Figure 6.**
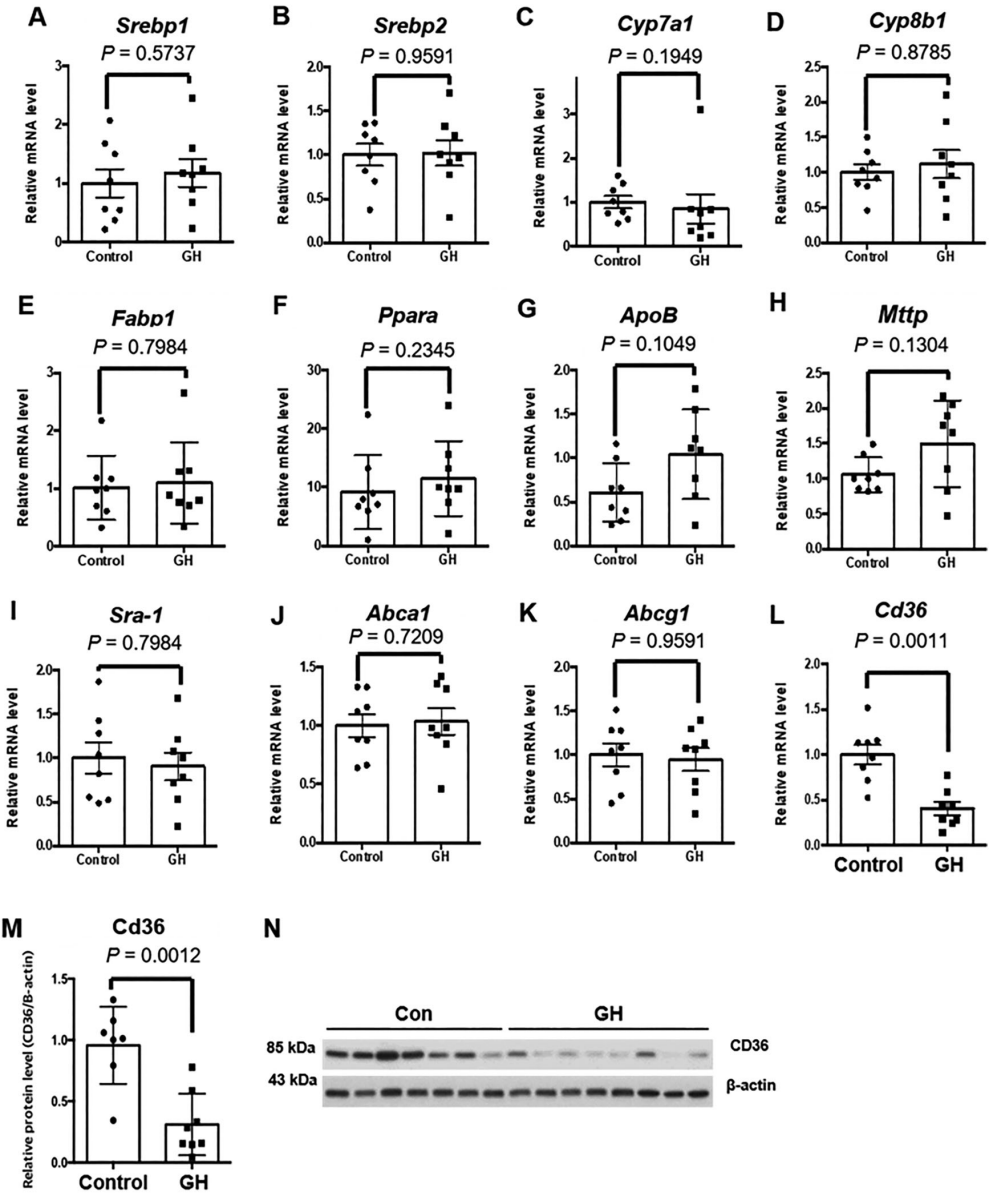
Effects of GH treatment on hepatic gene expressions related to lipid transport and metabolism. Hepatic gene expressions related to lipid transport and metabolism were analyzed by quantitative RT-PCR. (A) *Srebp1*, (B) *Srebp2*, (C) *Cyp7a1*, (D) *Cyp8b1*, (E) *Fabp1*, (F) *Ppara*, (G) *ApoB*, (H) *Mttp*, (I) *Sra-1*, (J) *Abca1*, and (K) *Abcg1* expressions in each group. There were no significant differences in these gene expressions between groups. (L) The hepatic mRNA level of CD36 was significantly decreased in the GH-treated group. (M, N) The decreased protein level of CD36 by GH treatment was further confirmed using western blot analysis.

**Figure 7.**
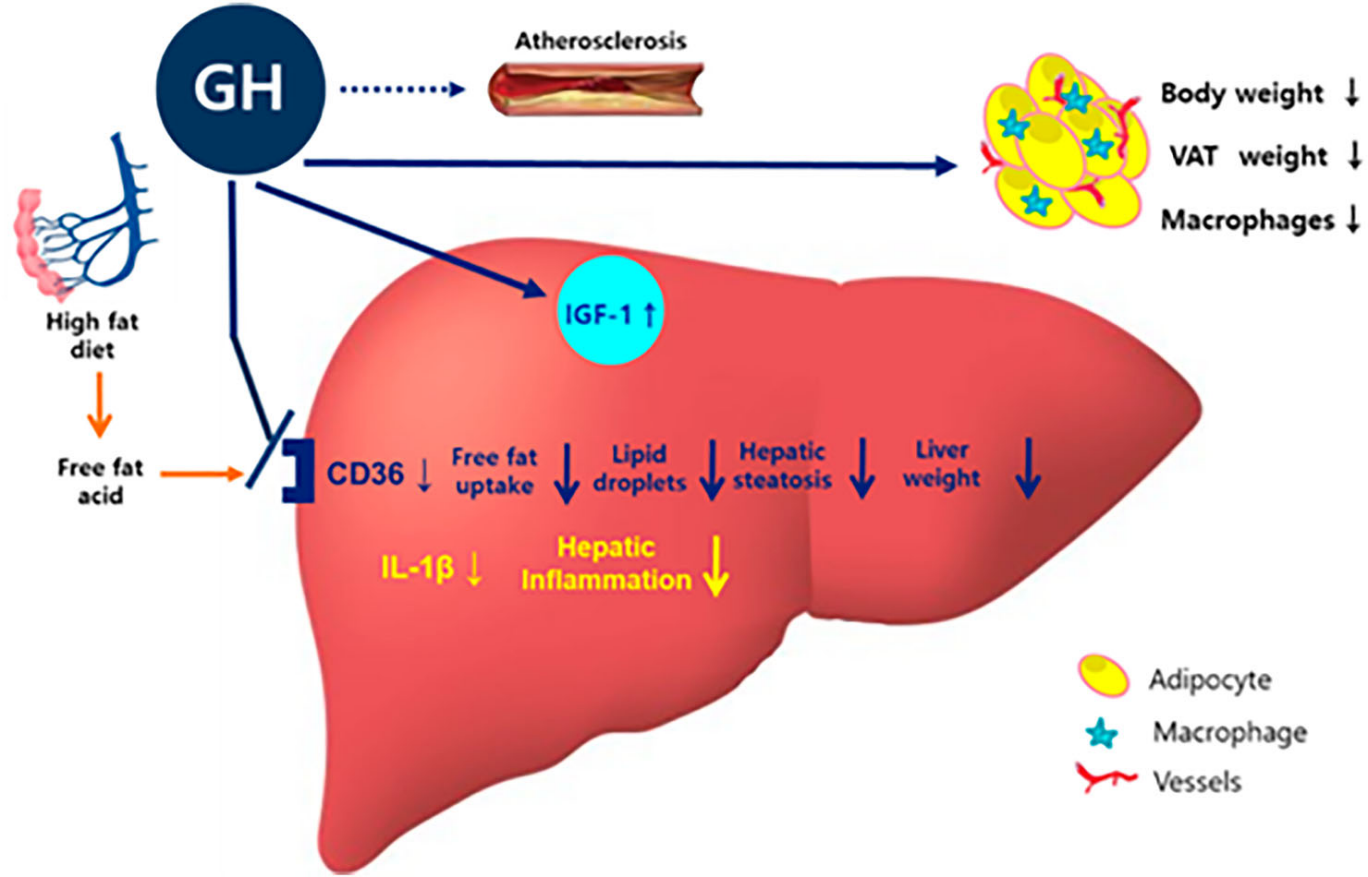
Schematic summary of the effect of GH treatment on the pathological changes in liver, adipose tissue, and aorta during the hyperlipidemic condition.

Next, the expression levels of hepatic genes related to cholesterol transport and metabolism were investigated in control and GH treated groups. The expression levels of Srebf1 and Srebf2 were not changed by GH treatment (Figure 6(A and B)). In addition, the mRNA levels of hepatic Cyp7a1, Cyp8b1, Fabp1, Ppara, ApoB, and Mttp did not differ between the two groups (Figure 6(C–H)). The mRNA levels of cholesterol transporters, namely, scavenger receptors (SR) A-1, ATP bind cassette (ABC) A1, and ABCG1, did not differ between the two groups (Figure 6(I–K)). However, the mRNA level of CD36, a key player for hepatic fatty acid uptake, was markedly decreased in the GH-treated group (Figure 6(L)). Moreover, the protein level of CD36 also decreased in GH treated group (Figure 6(M and N)). These results indicate that the decreased expression of hepatic CD36 may be responsible for the attenuation of hepatic steatosis and inflammation in GH treated mice. In summary, GH treatment inhibited inflammations in adipose tissue and liver, as well as hepatic steatosis, but had no effect on atherosclerosis in our study (Figure 7).

## Discussion

Exogenous GH treatment has been reported to reduce abdominal/visceral fat accumulation (Skaggs and Crist 1991), and GH promotes lipolysis and is used as a therapeutic agent to reduce total and abdominal fat masses. In addition, GH significantly reduces lipogenesis in adipose tissues, resulting in a significant loss of fat tissues (Rasmussen 2010). These previous reports are consistent with our data including significantly decreased weights of the body, liver, and VAT in the GH-treated group. However, a previous study has shown that GH reduces the levels of plasma cholesterol and triglycerides in *Ldlr^-/-^* mice, and their levels are further decreased by administration of GH (Rudling and Angelin 2001). Moreover, GH has been reported to increase LDL receptor expression, responsible for the lipid-lowering effect of GH (Rudling et al. 1992). However, in our study, we did not observe any significant changes in plasma lipid concentrations in GH treated mice. These discrepancies may be caused by different protocols for GH administration. Rudling et al. infused GH to normal chow diet fed *Ldlr^-/-^* mice at rate of 1 mg/kg/day using osmotic minipump. Thus, the dosage of GH used in our study might not be enough to regulate the genes related to cholesterol metabolism in Western-diet fed *Ldlr^-/-^* mice. But considering increased plasma IGF-1 concentration in our study, it appears that our protocol for GH treatment is effective in activating hepatocytes to produce IGF-1.

Previously, it has been known that functional receptors for GH and IGF-I are expressed in the endothelium of blood vessels (Delafontaine 1995). GHD is associated with low-grade chronic inflammation, endothelial dysfunction, and increased cardiovascular mortality. GH replacement therapy can improve low-grade chronic inflammation of atherosclerosis (Di Somma et al. 2017). In this study, there were no significant differences in lesion size, collagen content, and smooth muscle cell accumulation in atherosclerotic lesions between control and GH treated mice. Thus, it appears that the dosage of GH in our study was not effective to attenuate atherosclerotic progression. On the contrary, overexpression growth hormone exacerbated atherosclerosis in apolipoprotein E KO mice (Andersson et al. 2006). Therefore, further studies are required to define the exact effect of GH on atherosclerosis.

The prevalence of NAFLD is high in patients of hypopituitarism, and the severity of fatty liver was related to the concentration of serum GH, and the prevalence of NAFLD is closely related to GH deficiency (Adams et al. 2004; Hong et al. 2011). In the present study, GH treated group showed few lipid droplets in hepatocytes and less hepatic inflammation compared to the control group. Importantly, macrovesicular and microvesicular steatosis were significantly attenuated in the GH-treated group. CD36 (SR-B2), a class B scavenger receptor is a 88 kDa transmembrane glycoprotein receptor found in monocytes, macrophages, platelets, microvessel endothelial cells, adipocytes, and epithelial cells of the kidney and heart (Febbraio et al. 2001). CD36 level is increased by a high-fat diet, inflammation, and oxidative stress (Nishikawa et al. 2012). SR-A1 and CD36 act as a cholesterol influx transporter, whereas ABCA1 and ABCG1 act as a cholesterol efflux transporter. In this study, the mRNA levels of Sra1, Abca1, and Abcg1 were not significantly different between the two groups. However, the mRNA and protein levels of Cd36 in the liver was significantly reduced in the GH-treated group compared to the control. CD36 is associated with obesity and diabetes, and CD36-mediated hepatic fat uptake accelerates the progression of NAFLD. Thus, CD36 is suppressed by exogenous GH in an *Ldlr^-/-^* high-fat diet-fed mouse model, and it appears that the downregulated CD36 is responsible for attenuated hepatic steatosis by the GH treatment. We also investigated the changes in the mRNA levels of pro and anti-inflammatory genes and found that the *il1b* expression was markedly decreased in the GH treated group. Moreover, although there was no statistical significance, mRNA expression of IL-6 was also decreased by GH treatment. These results indicate that GH treatment is highly effective in attenuating hepatic steatosis and inflammation.

Taken together, exogenous GH decreased the weights of body, liver, and visceral adipose tissues, increased plasma IGF-1 concentration, reduced the recruitment of VAT macrophages, and decreased the hepatic expressions of IL-1β and CD36 in *Ldlr^-/-^* mice fed a high-fat diet. Our results suggest that GH treatment could be effective in the alleviation of inflammation and hepatic steatosis in hyperlipidemic conditions.

## References

[CIT0001] Adams LA, Feldstein A, Lindor KD, Angulo P. 2004. Nonalcoholic fatty liver disease among patients with hypothalamic and pituitary dysfunction. Hepatology. 39:909–914. doi: 10.1002/hep.20140 15057893

[CIT0002] Andersson IJ, Ljungberg A, Svensson L, Gan LM, Oscarsson J, Bergstrom G. 2006. Increased atherosclerotic lesion area in apoE deficient mice overexpressing bovine growth hormone. Atherosclerosis. 188:331–340. doi: 10.1016/j.atherosclerosis.2005.11.020 16368099

[CIT0003] Angulo P. 2002. Nonalcoholic fatty liver disease. N Engl J Med. 346:1221–1231. doi: 10.1056/NEJMra011775 11961152

[CIT0004] Binay C, Simsek E, Yıldırım A, Kosger P, Demiral M, Kılıç Z. 2015. Growth hormone and the risk of atherosclerosis in growth hormone-deficient children. Growth Horm IGF Res. 25:294–297. doi: 10.1016/j.ghir.2015.08.005 26296621

[CIT0005] Caicedo D, Diaz O, Devesa P, Devesa J. 2018. Growth Hormone (GH) and Cardiovascular System. Int J Mol Sci. 19:290. doi: 10.3390/ijms19010290 PMC5796235 29346331

[CIT0006] Chishima S, Kogiso T, Matsushita N, Hashimoto E, Tokushige K. 2017. The relationship between the growth hormone/insulin-like growth factor system and the histological features of nonalcoholic fatty liver disease. Intern Med. 56:473–480. doi: 10.2169/internalmedicine.56.7626 28250290 PMC5399195

[CIT0007] Delafontaine P. 1995. Insulin-like growth factor I and its binding proteins in the cardiovascular system. Cardiovas Res. 30:825–834. doi: 10.1016/S0008-6363(95)00163-8 8746194

[CIT0008] Di Somma C, Scarano E, Savastano S, Savanelli MC, Pivonello R, Colao A. 2017. Cardiovascular alterations in adult GH deficiency. Best Pract Res Clin Endocrinol Metab. 31:25–34. doi: 10.1016/j.beem.2017.03.005 28477729

[CIT0009] Febbraio M, Hajjar DP, Silverstein RL. 2001. CD36: a class B scavenger receptor involved in angiogenesis, atherosclerosis, inflammation, and lipid metabolism. J Clin Invest. 108:785–791. doi: 10.1172/JCI14006 11560944 PMC200943

[CIT0010] Fisker S, Vahl N, Hansen TB, Jørgensen JO, Hagen C, Orskov H, Christiansen JS. 1998. Growth hormone (GH) substitution for one year normalizes elevated GH-binding protein levels in GH-deficient adults secondary to a reduction in body fat. A placebo-controlled trial. Growth Horm IGF Res. 8:105–112. doi: 10.1016/S1096-6374(98)80100-7 10987677

[CIT0011] Graham MR, Evans P, Thomas NE, Davies B, Baker JS. 2009. Changes in endothelial dysfunction and associated cardiovascular disease morbidity markers in GH-IGF axis pathology. Am J Cardiovasc Drugs. 9:371–381. doi: 10.2165/11312100-000000000-00000 19929035

[CIT0012] Hashimoto E, Tokushige K. 2011. Prevalence, gender, ethnic variations, and prognosis of NASH. J Gastroenterol. 46(Suppl 1):63–69. doi: 10.1007/s00535-010-0311-8 20844903

[CIT0013] Hong JW, Kim JY, Kim YE, Lee EJ. 2011. Metabolic parameters and nonalcoholic fatty liver disease in hypopituitary men. Horm Metab Res. 43:48–54. doi: 10.1055/s-0030-1265217 20865648

[CIT0014] Juul A. 2003. Serum levels of insulin-like growth factor I and its binding proteins in health and disease. Growth Horm IGF Res. 13:113–170. doi: 10.1016/S1096-6374(03)00038-8 12914749

[CIT0015] Kargi AY, Merriam GR. 2013. Diagnosis and treatment of growth hormone deficiency in adults. Nat Rev Endocrinol. 9:335–345. doi: 10.1038/nrendo.2013.77 23629539

[CIT0016] Kelley KW, Weigent DA, Kooijman R. 2007. Protein hormones and immunity. Brain Behav Immun. 21:384–392. doi: 10.1016/j.bbi.2006.11.010 17198749 PMC1894894

[CIT0017] Nishikawa S, Sugimoto J, Okada M, Sakairi T, Takagi S. 2012. Gene expression in livers of BALB/C and C57BL/6J mice fed a high-fat diet. Toxicol Pathol. 40:71–82. doi: 10.1177/0192623311422078 22105644

[CIT0018] Rasmussen MH. 2010. Obesity, growth hormone and weight loss. Mol Cell Endocrinol. 316:147–153. doi: 10.1016/j.mce.2009.08.017 19723558

[CIT0019] Rasmussen MH, Frystyk J, Andersen T, Breum L, Christiansen JS, Hilsted J. 1994. The impact of obesity, fat distribution, and energy restriction on insulin-like growth factor-1 (IGF-1), IGF-binding protein-3, insulin, and growth hormone. Metabolism. 43:315–319. doi: 10.1016/0026-0495(94)90099-X 7511202

[CIT0020] Rudling M, Angelin B. 2001. Growth hormone reduces plasma cholesterol in LDL receptor-deficient mice. FASEB J. 15:1350–1356. doi: 10.1096/fj.00-0715com 11387232

[CIT0021] Rudling M, Norstedt G, Olivecrona H, Reihner E, Gustafsson JA, Angelin B. 1992. Importance of growth hormone for the induction of hepatic low density lipoprotein receptors. Proc Natl Acad Sci USA. 89:6983–6987. doi: 10.1073/pnas.89.15.6983 1495990 PMC49629

[CIT0022] Skaggs SR, Crist DM. 1991. Exogenous human growth hormone reduces body fat in obese women. Horm Res. 35:19–24. doi: 10.1159/000181870 1916649

[CIT0023] Takahashi Y, Iida K, Takahashi K, Yoshioka S, Fukuoka H, Takeno R, Imanaka M, Nishizawa H, Takahashi M, Seo Y, et al. 2007. Growth hormone reverses nonalcoholic steatohepatitis in a patient with adult growth hormone deficiency. Gastroenterology. 132:938–943. doi: 10.1053/j.gastro.2006.12.024 17324404

